# CRISPR editing of candidate host factors that impact influenza A virus infection

**DOI:** 10.1128/spectrum.02627-24

**Published:** 2025-01-31

**Authors:** Pyae Phyo Kyawe, Ping Liu, Zhaozhao Jiang, Evan S. Bradley, Thomas Cicuto, Melanie I. Trombly, Neal Silverman, Katherine A. Fitzgerald, William M. McDougall, Jennifer P. Wang

**Affiliations:** 1 Department of Medicine, Diabetes Center of Excellence, University of Massachusetts Chan Medical School, Worcester, Massachusetts, USA; 2 Interdisciplinary Graduate Program, Morningside Graduate School of Biomedical Sciences, Worcester, Massachusetts, USA; 3 Department of Medicine, Division of Innate Immunity, University of Massachusetts Chan Medical School, Worcester, Massachusetts, USA; 4 Department of Microbiology, University of Massachusetts Chan Medical School, Worcester, Massachusetts, USA; 5 Division of Infectious Diseases and Immunology, Department of Medicine, University of Massachusetts Chan Medical School, Worcester, Massachusetts, USA; Barnard College, Columbia University, New York, USA

**Keywords:** influenza, CRISPR, B4GALNT2, CMAS, ADAR1, host factors

## Abstract

Influenza A virus (IAV) is a respiratory pathogen with a segmented negative-sense RNA genome that can cause epidemics and pandemics. The host factors required for the complete IAV infectious cycle have not been fully identified. Here, we examined three host factors for their contributions to IAV infectivity. We performed CRISPR-mediated knockout of cytidine monophosphate N-acetylneuraminic acid synthetase (CMAS) as well as CRISPR-mediated overexpression of beta-1,4 N-acetylgalactosaminyltransferase 2 (B4GALNT2) and adenosine deaminase acting on RNA 1 (ADAR1) in the human bronchial epithelial A549 cell line and evaluated the impact on IAV and other RNA viruses. We confirmed that knockout of *CMAS* or overexpression of *B4GALNT2* restricts IAV infection by diminishing binding to the cell surface but has no effect on vesicular stomatitis virus infection. Although ADAR1 overexpression does not significantly inhibit IAV replication, it has a pro-viral effect with coxsackie B virus (CVB) infection. This pro-viral effect is not likely secondary to reduced type I interferon (IFN) production, as the induction of the IFN-stimulated genes *ISG15* and *CXCL10* is negligible in both parent and ADAR1-overexpressing A549 cells following CVB challenge. In contrast, *ISG15* and *CXCL10* production is robust and equal for parent and ADAR1-overexpressing A549 cells challenged with IAV. Taken together, these data provide insights into how host factors can be further explored to understand the dynamics of pro- and anti-viral factors.

**IMPORTANCE:**

Influenza A virus (IAV) remains a global threat due to its ability to cause pandemics, making the identification of host factors essential for developing new antiviral strategies. In this study, we utilized CRISPR-based techniques to investigate host factors that impact IAV infectivity. Knockout of CMAS, a key enzyme in sialic acid biosynthesis, significantly reduced IAV binding and infection by disrupting sialic acid production on the cell surface. Overexpression of B4GALNT2 had similar effects, conferring resistance to IAV infection through diminished cell-surface binding. Overexpression of ADAR1, known for its role in RNA editing and immune regulation, impacted IAV replication minimally but enhanced coxsackie B virus replication. Such findings reveal the diverse roles of host factors in viral infection, offering insights for targeted therapeutic development against IAV and other pathogens.

## INTRODUCTION

Influenza A virus (IAV) is an enveloped virus with a segmented negative-sense single-stranded RNA genome that is a recurring respiratory pathogen in humans. Understanding how IAV infects cells and identifying the host components necessary for productive infection is essential, given its tremendous potential for global illness, such as with pandemic outbreaks (1). The 2009 influenza pandemic and the recent SARS-CoV-2 pandemics showed how the spread of respiratory pathogens is driven by socioeconomic issues and can overwhelm the healthcare system; therefore, a finer understanding of IAV-host dynamics is warranted.

RNA-based genome-wide screening methods, mainly arrayed small interfering RNA (siRNA) and pooled short hairpin RNA (shRNA)-based screens, have been used to identify essential host factors necessary for productive IAV infection (2–7), but inconsistencies between studies are problematic (reviewed in [8]). CRISPR-Cas9 systems minimize the drawbacks of using RNAi-based systems as they have fewer off-target effects and allow for the generation of permanent homozygous null lines (9–11), therefore have become a standard format for genome-wide screens for host factors during virus infections due to ease of scalability and minimal off-target effects. CRISPR-based genome-wide knockout (KO) screens have been performed for viruses, including IAV (12–14), coronaviruses (15–17), flaviviruses (18–20), and HIV (21–23). Han et al. first utilized the CRISPR-Cas9-based gene KO screening strategy in A549 cells to determine host factors essential for the infection of avian influenza virus (13). Next-generation sequencing of survivor cells identified enrichment of host factors involved in sialic acid biosynthesis (including *CMAS* encoding cytidine monophosphate N-acetylneuraminic acid synthetase), sialic acid transport (*SLC35A1, SCL35A2* encoding members of solute carrier family 35), and glycan modification/processing (including *B4GALNT4* encoding beta-1,4-N-acetyl-galactosaminyltransferase 4) (13). Using a CRISPR-Cas9-based screen, Yi et al. similarly identified several sialic acid-related genes, including *SLC35A1, GNE, CMAS,* and *NANS*, as being critical for IAV infection (24). However, a downside of CRISPR-Cas9’s ability to generate a homozygous null phenotype also means that genes required for viability and proliferation are eliminated from the screen, since cells with such KO genes would not have survived or expanded. Candidates identified are those that confer strong resistance to virus infection, but survivor screens are not designed to detect candidates that confer moderate resistance to virus infection. Furthermore, pro-viral host factors will not be detected in a survivor screen.

The development of catalytically inactive, endonuclease-deficient Cas9 (dCas9) fused to transcriptional repressor domains, such as KRAB, permits the further study of gene function without generating a potential cell lethal phenotype. Furthermore, manipulating genes at the transcriptional level rather than generating indels on the genomic DNA results in fewer off-target effects, thereby decreasing the chances of false positives or negatives (25, 26). dCas9 can be fused to transcriptional activators (e.g., VPR, VP64, p65, and HSF) to induce overexpression through guide RNA (gRNA)-directed transcriptional expression instead of conventional cDNA-mediated overexpression (27, 28). A CRISPR-activation (CRISPRa) screen revealed that overexpression of *B4GALNT2* (encoding beta-1,4-N-acetyl-galactosaminyltransferase 2, a participant in sialic acid modification) results in inhibitory activity against influenza viruses with α2,3-linked sialic acid receptor preferences (29).

ADAR1 plays an important role in post-transcriptional editing of RNA, binding endogenous double-stranded (ds) RNA and catalyzing the hydrolytic deamination of adenosine to inosine. This prevents excessive innate immune responses to dsRNA mediated by the RIG-I-like receptor (RLR) melanoma differentiation-associated protein 5 (MDA5), specifically type I interferon (IFN) production and interferon-stimulated gene (ISG) induction (30). Missense or frameshift mutations in ADAR1 lead to fatal neurodegenerative disorders, such as Aicardi-Goutières syndrome, which is characterized by elevated ISGs due to the failure of the host to discriminate self from non-self dsRNA (31). ADAR1 has been proposed to have both pro- and anti-viral functions depending on the cell lines and viruses tested (32). Two isoforms of ADAR1 exist, the IFN-inducible cytoplasmic p150 and the constitutive nuclear p110. The role of the IFN-induced p150 isoform during IAV infection has been examined previously. Ward et al. initially reported that the ADAR1 p150 isoform protected host cells from IAV-induced cytopathic effects (33). In a later study, Vogel et al. showed that deletion of ADAR1 p150 resulted in sustained RLR signaling and increased apoptosis (34).

To explore host factors that confer resistance to virus infection as well as pro-viral host factors, we examined the role of *CMAS* in IAV infection. We also demonstrated how Cas9-driven overexpression of *B4GALNT2* using CRISPRa resulted in impaired sialic acid production accompanied by a reduction in IAV infection. Finally, we examined the impact of overexpressing isoforms of *ADAR1* (adenosine deaminase acting on RNA 1) in the context of IAV and other virus infections.

## MATERIALS AND METHODS

### Viruses

IAV strain WSN/33 (H1N1) was obtained from A. L. Brass (3), strain Puerto Rico/8/1934 (PR/8) and Hong Kong/1968 (HK/68) from Charles River Laboratories, and California/04/2009 (pdm2009) from American Type Culture Collection (ATCC, #VR-1805). VSV strain Indiana was obtained from ATCC (#VR-1238). CVB4 strain JVB was obtained from ATCC (#VR-184).

### Cell lines

The following cell lines were obtained from ATCC: A549 (#CCL-185), Madin-Darby canine kidney (MDCK) cells (#CCL-34), HeLa (#CCL-2), and baby hamster kidney (BHK-21) cells (#CCL-10). Cells were grown in Dulbecco’s Modified Eagle Medium (DMEM) supplemented with 10% fetal bovine serum, 100 U/mL penicillin, 100 µg/mL streptomycin, and GlutaMax. The cell lines were routinely monitored for mycoplasma. HEK293T and HEK293T ADAR1 KO cells were a gift from Dr. Daniel Stetson (35).

A549 Cas9 and A549-dCas9-VP64 cells were generated via lentiviral transduction of SpCas9 or the VP64 activator domain (dCas9-VP64) into A549 cells. Clonal cell lines with a high expression of Cas9 or dCas9-VP64 proteins were established by limiting dilution after selection with 10 µg/mL blasticidin S HCl (ThermoFisher #A1113903) for 14 days. A549 CMAS-deficient cell lines were generated by transfecting A549-Cas9 cells (36) with pGS-CMAS-Neo plasmid with sgRNA targeting exon 4 of CMAS with CCATCCCAGTCTTGTCGACG gRNA from a U6 promoter and a neomycin-resistance marker (GenScript). Clonal A549 CMAS-deficient cell lines were established by limiting dilution after selection with 800 µg/mL G418 (Geneticin, ThermoFisher #10131027) for 14 d.

For A549 Cal^B4GALNT2^ and Cal^ADAR1^ overexpressing lines (cell lines were named “Cal” for the Calabrese CRISPR activation library), sgRNA sequences for the CRISPRa candidate genes were cloned into expression vector pXPR-502 as described previously (28, 37). sgRNA targeting the B4GALNT2 promoter region or ADAR1 and complementary oligos with appropriate nucleotide overhangs (sgRNA for B4GALNT2: GGGCAAATTCTCGGCGAGTA, ADAR1 g1: AACCGGCCTGAAACCAAGCG, g2: CTTCCGTAGTTCTCATGCAG, g3: CGCTGCATGAGAACTACGGA) were ordered from ThermoFisher and annealed with a thermocycler to form dsDNA duplexes. Specifically, oligos were mixed with T4 Ligase Buffer (NEB) and T4 PNK enzyme, and annealing was performed using a thermocycler, following the program: 37°C for 30 min, 95°C for 5 min; and then decreasing the temperature by 5 °C/min until 25°C was reached. In addition, 90 µL of water was added to each reaction, and 2 µL of duplexed oligos was ligated to 25 ng of linearized pXPR-502 lentiviral expression vector in 20 µL ligation reaction at 16°C overnight. Seven microliters of the ligated material were used to transform Stbl3 *E. coli*, which was plated on LB plates containing 100 µg/mL ampicillin. At least three bacterial colonies were sent out for Sanger sequencing, and plasmids with the correct sgRNA sequences were expanded for lentiviral transduction (37). Confluent HEK293T cells in a 10 cm dish were transfected with plasmids expressing VSV-G envelope protein (pMD2.G), HIV structural proteins (psPAX2), and pXPR-502 expressing the specific sgRNA from a U6 promoter and a puromycin resistance gene from an EF-1a promoter at the ratio of 1:1.5:3 (4, 6, and 12 µg, respectively) using TransIt-293T. The supernatants were collected every 24 h for 3 days and pooled. The supernatant was concentrated with LentiX-concentrator and added to A549-dCas9-VP64 cells. Lentivirus-transformed cells were then selected with 2 µg/mL puromycin for 7 days. The survivor cells were then expanded and maintained in complete DMEM supplemented with 2 µg/mL puromycin. Supernatants were collected for 3 days and passed through a 0.45 µm filter to remove debris before being added to target cell lines. Media was replaced with DMEM + 10 µg/mL blasticidin and 2 µg/mL puromycin (A1113803, Invitrogen) at 24 h post-transduction, and the cells were cultured for 7 days. Clonal knockdown and overexpression cell lines were established by limiting dilution and with passage in selective media.

### Western blots

Cells were lysed with radioimmunoprecipitation assay (RIPA) buffer with protease inhibitor cocktail (Sigma #P8340) for 10 min at room temperature. The cell lysate was centrifuged at 20,000 × *g* for 10  min to remove cell debris. The concentration of the lysate was determined by bicinchoninic acid protein assay (BCA assay) (ThermoFisher #PI23227). Thirty-five micrograms of protein lysate were heated at 95°C for 5 min and loaded onto Novex Tris-Glycine Mini-Protein Gel 4%–12% (ThermoFisher #XP04200BOX) and run at 100 V for 1.5 h. The proteins were then transferred onto a polyvinylidene fluoride (PVDF) membrane (ThermoFisher #88518) with an Xcell II Blot Module (ThermoFisher #EI0002) for 1 h at 20 V. The membrane was blocked with 5% nonfat dry milk in Tris-buffered saline (TBS) + 0.1% Tween 20 (TBS-T) for at least 1 h. The membrane was incubated with primary antibody—mouse anti-CMAS (Millipore Sigma #SAB1405174) diluted 1:1,000 in 5% nonfat dry milk in TBS-T or rabbit anti-ADAR (Cell Signaling #81284S) diluted 1:1,000 in 5% nonfat dry milk/TBS-T—overnight with constant rocking. Membranes were washed three times with TBS-T and incubated with horseradish peroxidase (HRP)-conjugated secondary antibody—horse anti-mouse IgG-HRP (Cell Signaling #7076S) diluted 1:1,000 in 5% nonfat dry milk/TBS-T or goat anti-rabbit IgG-HRP (Vector Laboratories #PI-1000) diluted 1:20,000 in 5% nonfat dry milk/TBS-T—for 1 h at room temperature. Membranes were washed five times with TBS-T and then visualized with SuperSignal West Pico PLUS Chemiluminescent Substrate (ThermoFisher #34580) using a BioRad Imager. Membranes were stripped with Restore Plus Western stripping buffer (ThermoFisher #46430) for 10 min and washed twice with TBS-T. The membrane was then blocked in 3% bovine serum albumin (BSA) in TBS-T for 1 h and then incubated with anti-actin HRP (Santa Cruz Biotechnology #sc-37778) diluted 1:2,000 in 3% BSA for 1 h. The membrane was washed five times in TBS-T and visualized.

### RT-qPCR

RNA was extracted from cell lines with TRIzol, and 1 µg of RNA was converted to cDNA using the Qiagen reverse transcription kit. Equal concentrations of cDNA were loaded for the quantification of target gene levels by SYBR Green qPCR using HPRT as a housekeeping gene for 40 cycles. Fold change was calculated based on the 2^ΔΔCT^ method. Primers are as follows (Life Technologies Corporation, ThermoFisher):

*CMAS* F: ACAAGACTGGGATGGAGAA, R: ACTATGTTCAGCTCGCATTT; *B4GALNT2* F: CTACGATGGAATCTGGCTGTT, R: GCCATAGGCATCCTGAAAGT; *ADAR1* F: ATCAGCGGGCTGTTAGAATATG, R: AAACTCTCGGCCATTGATGAC; *HPRT* F: ATCAGACTGAAGAGCTATTGTAATGA, R: TGGCTTATATCCAACACTTCGTG; *ISG15* F: CGCAGATCACCCAGAAGATCG, R: GATCTCAGAAATACCCCAGCCA; and *CXCL10* F: TTCTGATTTGCTGCCTTATCTTTC, R: TTCTTGATGGCCTTCGATTCTGG.

### RNA-sequencing and analysis

RNA was purified from cell lines by TRIzol Reagent. Strand-specific total RNA with ribosomal RNA depletion (1 µg of input RNA) was used to generate libraries generated with the TruSeq Stranded Total RNA Sample Prep Kit (Illumina) and sequenced on an Illumina NextSeq 2000 machine. Paired-end sequence reads were aligned to the human reference genome (hg38_v44) using STAR (38), and transcript quantification was performed with RSEM (39) through the UMass Chan Medical School DolphinNext (40). Downstream analysis was performed in R version 4.4.0 using the RStudio interface. Relative transcript abundance was estimated using DESeq2 based on normalized counts of alignments, three replicates of parental A549-dCas9-VP64 were compared with clones overexpressing either ADAR1 or B4GALNT2 (41). The cutoff for significance was *P* < 0.1 adjusted for multiple comparisons. Volcano plots were constructed using the EnhancedVolcano package (42). The scRNA-seq raw fastq files, gene by cell transcript counts matrix, and metadata file are deposited in the NCBI GEO database with accession number GSE284495.

### Lectin staining and flow cytometry

Cells were trypsinized at 37°C for 5 min to obtain single-cell suspensions and transferred to round bottom 96-well plates at 2 × 10^5^ cells per well. Cells were washed with 200 µL of phosphate-buffered saline (PBS) twice. Biotinylated lectins *Maackia amurensis* (MAA, 2 µg/mL, Vector Laboratories #B-1265–1), *Sambucus nigra* (SNA, 2 µg/mL, Vector Laboratories #B-1305–2), or *Dolichos biflorus* agglutinin DBA (2 µg/mL, Vector Laboratories #B-1035–5) were added to each well in 100 µL volume and binding was performed for 1 h at 4°C. Cells were washed twice with PBS to remove the unbound lectins and Alexa Fluor 647-conjugated streptavidin (SA-647, ThermoFisher Scientific #S21374) was added to each well at a working concentration of 2 µg/mL for 1 h at 4°C. The cells were washed twice with PBS and fixed with 4% paraformaldehyde (PFA) for 10 min at 4°C and then washed twice with PBS and resuspended in 150 µL PBS for flow cytometry. At least 10,000 events were captured and analyzed on a BD FACSCelesta running BD FACS Diva Software with a standard laser and filter combination. All data were visualized and analyzed with FlowJo software.

### IAV infection and TCID_50_ assay

Cells were plated at 1.5 × 10^4^ cells/well in 100 µL media in 96-well black plates with a clear bottom (Corning #3603) and incubated overnight at 37°C. The cells were then challenged with virus diluted in 100 µL serum-free DMEM for 1 h at 37°C. Cells were washed to remove unbound virus, and 100 µL of fresh growth media was added to each well. Plates were incubated for 24 h at 37°C, and the supernatants were collected and saved at −80°C for 50% tissue culture infectious dose (TCID_50_) assays. Cells were fixed in 4% PFA for 15 min at room temperature and permeabilized with 0.2% Triton X-100 in PBS for 15 min at room temperature. The cells were then stained with anti-HA antibody at 1:20 (hybridoma HA36-4-5.2, Wistar Institute), followed by Alexa Fluor 488 goat anti-mouse secondary antibody at 1:1,000 (A11001, Invitrogen) or Alexa Fluor 647 goat anti-mouse secondary antibody at 1:1,000 (A-21235, Invitrogen). For some experiments, anti-NP antibody (Millipore Sigma, MAB8258, 1:1,000 in PBS + 1% BSA), followed by goat anti-mouse IgG-conjugated to Alexa Fluor 647 (ThermoFisher #A21235 1:1000 in PBS + 1% BSA) was used. Cells were imaged using the Celigo Imaging Cytometer (Nexcelom) and HA- or NP-positive cells normalized to the total number of cells (determined by DAPI, Invitrogen #D1306) were analyzed using the “Expression Analysis” on the Celigo Imaging Cytometer.

For multi-cycle viral replication assays, the cells were seeded in 24-well plates and infected, and the supernatants were collected at 1, 24, and 48 h post-infection (hpi) and stored at −80°C. Samples were titered on MDCK cells using a standard TCID_50_ assay as described elsewhere (43). In brief, MDCK cells were plated at 2 × 10^5^ cells/well in 96-well plates in 100 µL growth media and incubated overnight at 37°C. The supernatants were thawed on ice and 10-fold serial dilutions of viruses were prepared in serum-free DMEM. MDCK cells were washed twice with PBS to remove FBS, and then, 100 µL of diluted viruses was added to MDCK cells in replicates of eight. The cells were then incubated at 37°C for 1 h with gentle shaking. Virus dilutions were removed by aspiration, and the cell monolayers were then washed twice with PBS to remove the unbound virus. Cells were then maintained in DMEM supplemented by 0.2% BSA + 0.5 µg/mL tosyl phenylalanyl chloromethyl ketone (TPCK)-treated trypsin (Sigma #T8802) and monitored for cytopathic effect daily. After 72 h, the TCID_50_/mL was calculated using the Reed-Muench method. For some experiments, the virus was titered by plaque assay using MDCK cells as previously reported (44).

### Sialidase treatment and IAV infection of A549 cells

A549 cells (2 × 10^4^ cells/well) were seeded in 96-well plates and incubated overnight at 37°C. The cells were treated with various concentrations of *C. perfringens* sialidase (Sigma Aldrich #11585886001) for 1 h at 37°C, washed, then infected with IAV WSN/33 (MOI 50) for 8 h. The percentage of NP+ cells was determined by NP immunofluorescence normalized to total cell count (nuclear staining).

### VSV infection and plaque assay

Cells were plated and infected with VSV, then the supernatants were collected 24 hpi and titered on BHK-21 cells as described previously (45). Ten-fold serial dilutions of supernatants in serum-free DMEM were added to confluent BHK-21 cells in 12-well plates in a final volume of 100 µL/well in duplicates. The cells were then incubated at 37°C for 1 h with gentle shaking. Supernatants were removed by aspiration, and the cell monolayers were washed with serum-free DMEM. A 2% agarose overlay with 1× Modified Eagle’s Medium (Gibco #11935) was added to each well and allowed to solidify at room temperature and then incubated at 37°C for 24 h. Cells were fixed/stained for at least 2 h at room temperature with 0.5% crystal violet dissolved in 4% PFA. Overlays were removed, and the PFU/mL was calculated by averaging the number of plaques in the duplicate wells and dividing by the product of the dilution factor and volume added to each well.

### CVB infection and plaque assay and poly I:C treatment

The cells were plated and infected with CVB4, and then, the supernatants were collected at 24 hpi and titered on HeLa cells based on standard protocols. Ten-fold serial dilutions of supernatants in serum-free DMEM were added to confluent HeLa cells in 6-well plates in a final volume of 100 µL/well in duplicates. Cells were then incubated at 37°C for 1 h with gentle shaking. Supernatants were removed by aspiration, and the cell monolayers were washed with serum-free DMEM. An overlay mixture (1% agar, 1× Modified Eagle’s Medium, 5% FBS, and 5 mM MgCl_2_) was added to each well and allowed to solidify at room temperature and then incubated at 37°C for 48 h. Five hundred microliters of 2× MTT/INT dye (thiazolyl blue tetrazolium bromide/iodonitrotetrazolium chloride) were added on top of each overlay, and the plates were incubated at 37°C for at least 2 h. PFU/mL was calculated by averaging the number of plaques in the duplicate wells and dividing by the product of the dilution factor and volume added to each well. High-molecular-weight (HMW) poly I:C (Invivogen #tlrl-pic) was added to cells for 24 h.

### Fluorescence *in situ* hybridization

The cells were seeded onto rat tail collagen-coated coverslips in 12-well plates at 10,000 cells/well in 1 mL complete DMEM overnight. Cells were then washed twice with serum-free DMEM and prechilled on ice for 45 min. IAV WSN/33 diluted in DMEM/0.2% BSA was added to each well at an MOI of 1, and binding was performed on ice for 1 h. Unbound virus was washed off with serum-free DMEM and pre-warmed DMEM + FBS was added to each well to facilitate endocytosis and cells were kept at 37°C for 20 min. The cells were fixed with 4% PFA for 10 min at room temperature and permeabilized with 70% ethanol for 2 h at 4°C. Hybridization was performed using Quasar 570-conjugated fluorescence *in situ* hybridization (FISH) probes against segment 7 of WSN/33 viral RNA (vRNA, sequences available at [46]) following the manufacturer’s protocol (BioSearch Technologies). Briefly, the cells were washed with Wash Buffer A for 5 min, and then, a fluorescence *in situ* hybridization (FISH) probe in hybridization buffer was added for 12 h at 37°C. The probes were removed by washing the cells with Wash Buffer A for 30 min at 37°C in the dark. DAPI (5 ng/mL diluted in Wash Buffer A) was added to each cell and stained in the dark for 30 min at 37°C. DAPI was removed, and the cells were incubated in Wash Buffer B for 5 min at room temperature. Coverslips were mounted with ProLong Diamond Antifade Mountant (ThermoFisher #P36970). ImageJ was used to determine the average mean fluorescence intensity (representing the average intensity of WSN/33 segment 7 vRNA staining) in each cell.

### IAV-binding assay

Trypsinized cells were added to 96-well round bottom plates at 2 × 10^5^ cells per well and chilled on ice for 1 h at 4°C. Cells were washed with cold PBS, and then, IAV WSN/33 was added to the prechilled cells at the indicated MOI in 100 µL serum-free DMEM. IAV binding was performed on ice for 1 h. Cells were washed twice with cold PBS to remove unbound virus and then fixed with 4% PFA for 10 min at 4°C. Cells were then washed with PBS twice before staining with anti-HA antibody (hybridoma HA36-4-5.2, Wistar Institute, 1:100 dilution in PBS/1% BSA) for 1 h at 4°C. Cells were then washed with PBS twice and stained with Alexa Fluor 647-conjugated goat anti-mouse antibody (ThermoFisher #A21235) diluted 1:400 in PBS/1% BSA for 1 h at 4°C. Cells were then washed twice with PBS and resuspended in 150 µL PBS for flow cytometry.

### Microscopy

The samples were imaged by confocal microscopy using a Leica SP-8 Stellaris System.

### Statistical analysis

Statistical analyses were performed using GraphPad Prism Version 10.1.1. Data are presented as mean ±  standard deviation for at least three independent experiments. Statistical tests were used as indicated in the figure legends. *P* < 0.05 was considered statistically significant.

## RESULTS

### Targeting of CMAS by CRISPRi in A549 cells inhibits infection with IAV but not VSV

**Fig 1:**
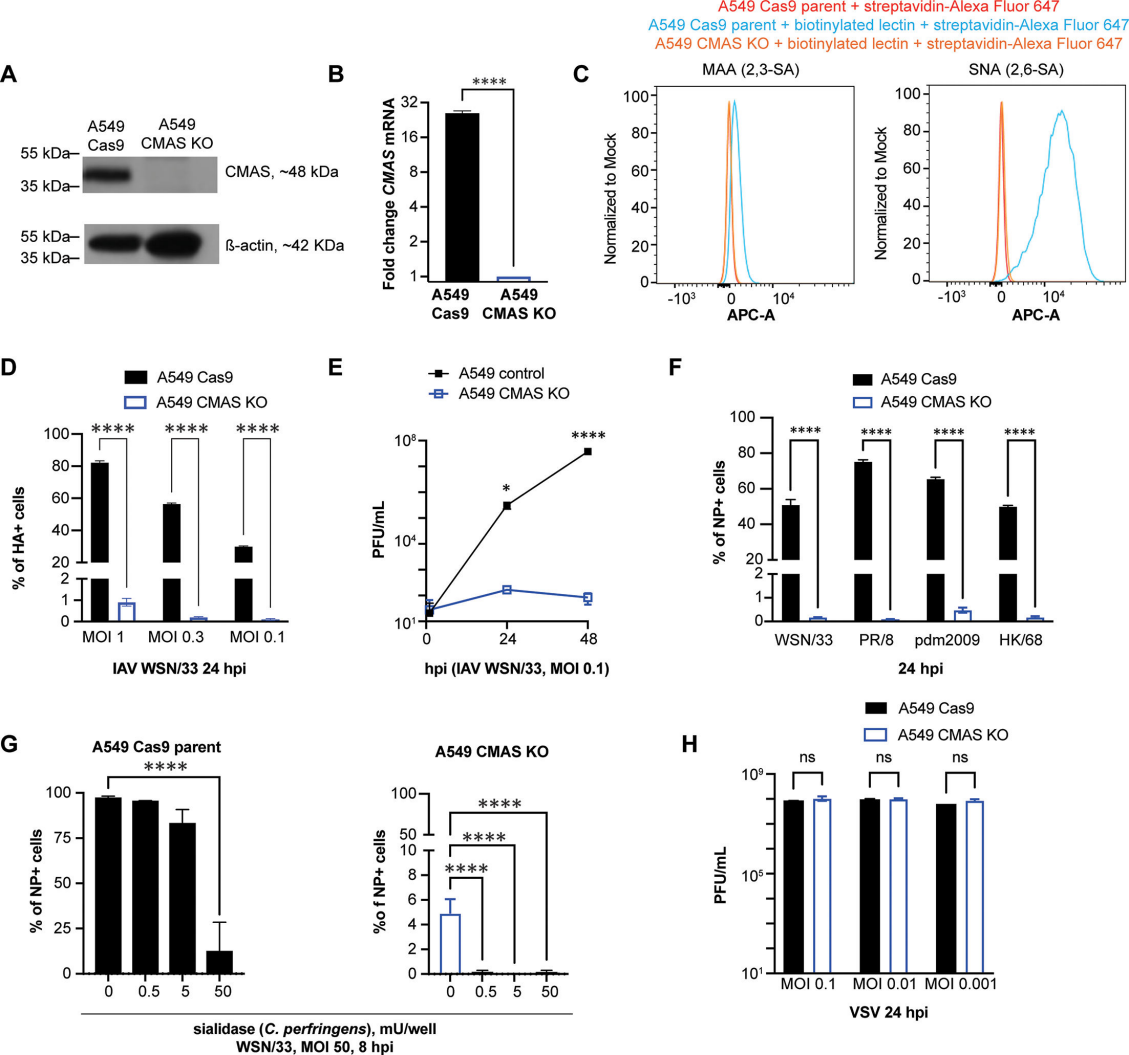
IAV infection is reduced, whereas VSV infection is sustained in A549 CMAS-deficient cells. (A) Western blot from clonal A549 CMAS-deficient cells show the loss of CMAS protein. Actin is included as a loading control.** (B).** *CMAS* expression in A549 CMAS KO cells is reduced compared with parent A549 Cas9 cells by RT-qPCR. RNA was extracted from each cell line and reverse transcribed. *CMAS* and *HPRT* (housekeeping gene) were amplified, and the *CMAS* fold change was calculated based on the 2^ΔΔCT^ method. ****, *P* < 0.0001, unpaired *t*-test.** (C)** Flow cytometry histograms of binding of biotinylated SNA lectin (specific to α2,6-linked sialic acid), biotinylated MAA lectin (specific to α2,3-linked sialic acid), and streptavidin-Alexa Fluor 647 to A549 Cas9 and A549 CMAS KO cell lines. Each group has triplicate samples and a representative sample from each group is shown. (**D)** A549 Cas9 parent and A549 CMAS KO cells were infected with IAV WSN/33 with the indicated MOI. At 24 hpi, the percentage of HA + cells was determined by anti-HA +staining normalized to DAPI staining. ****, *P* < 0.0001, two-way ANOVA.** (E)** A549 and A549 CMAS KO cells were infected with IAV WSN/33 at the indicated MOI. Supernatants were collected at 1, 24, and 48 hpi, and plaque-forming units (PFU) were determined. *, *P* < 0.01, ****; *P* < 0.0001, unpaired *t*-test. (**F)** A549 Cas9 parent and A549 CMAS KO cells were infected with IAV WSN/33 (H1N1), PR/8 (H1N1), pdm2009 (H1N1), and HK/68 (H3N2) using an inoculum at which ≥ 40% of A549 Cas9 cells are NP +at 24 hpi (normalized to DAPI). ****, *P* < 0.0001, two-way ANOVA.** (G)** IAV infection is decreased in both parent and CMAS KO A549 cells following sialidase treatment. Cells were treated with up to 50 mU sialidase per well for 1 h, washed, and then infected with IAV WSN/33 at MOI 50. At 8 hpi, the percentage of NP+ cells was determined. ****, *P* < 0.0001, one-way ANOVA.** (H).** A549 Cas9 parent and A549 CMAS KO cells were infected with VSV at the indicated MOI. Supernatants were collected at 24 hpi, and the PFU/mL was determined. ns = not significant, two-way ANOVA. Error bars represent the S.D. for triplicate samples.

CMAS converts N-acetylneuraminic acid to cytidine-5-monophosphate (CMP)-sialic acid in the nucleus (13, 47) and has been shown to be important for attachment and entry of swine, avian, and human IAVs (48). To confirm the role of CMAS during IAV infection, we generated clonal CMAS KO cells by transducing specific sgRNAs in A549-Cas9 cells and performing limiting dilution post-G418 selection. Since CMAS is the rate-limiting enzyme in sialic acid biosynthesis, we tested for loss of CMAS function by staining cells with *Sambucus nigra* lectin (SNA), a lectin that specifically binds to α2,6-linked sialic acid on the cell surface, and performing flow cytometry. Five to 10 clonal lines were initially screened for loss of SNA staining. The clonal line with the lowest amount of cell surface sialic acid was selected for further validation and infection experiments. Reductions in CMAS protein and *CMAS* mRNA were confirmed by western blot and RT-qPCR (Fig. 1A and B). SNA binding was abundant in parent A549 cells and markedly decreased in CMAS-deficient cells. Staining with the α2,3-linked sialic acid-binding *Maackia amurensis* lectin (MAA) is less abundant than with SNA in parent A549 cells yet is also reduced in CMAS-deficient cells (Fig. 1C).

To test how CMAS deficiency impacts IAV infection, A549-Cas9 and CMAS KO cells were challenged with IAV WSN/33 and then fixed and stained with an anti-hemagglutinin (HA) antibody at 24 hpi. IAV WSN/33 infection was significantly reduced in CMAS KO compared with parent cells (Fig. 1D). To confirm that multi-cycle viral replication was also impacted, infected cells were monitored over 48 h with supernatant collected at 1, 24, and 48 hpi (Fig. 1E). CMAS KO reduced infection of several IAV strains, including PR/8 (H1N1), pdm2009 (H1N1), and IAV strain HK/68 (H3N2) to similar degrees (Fig. 1F).

Since the CMAS KO cells showed evidence of residual IAV infection, we aimed to determine if sialidase treatment further reduces IAV infection. CMAS KO A549 cells were treated with several doses of sialidase prior to infection. Although sialidase treatment did not impact cell adhesion or result in cell toxicity, it resulted in a significant reduction in IAV entry based on NP staining at 8 hpi in both parent and CMAS KO A549 cells (Fig. 1G).

Finally, to determine if the inhibition was specific for IAV, CMAS KO, and parent A549 cells were challenged with vesicular stomatitis virus (VSV) at the indicated MOI. Virus in supernatants collected at 24 hpi was titered by plaque assay on BHK-21 cells. CMAS KO did not significantly impact VSV infection, as comparable levels of virus were recovered from supernatants (Fig. 1H).

### Overexpression of the B4GALNT2 inhibits infection with IAV but not VSV

**Fig 2:**
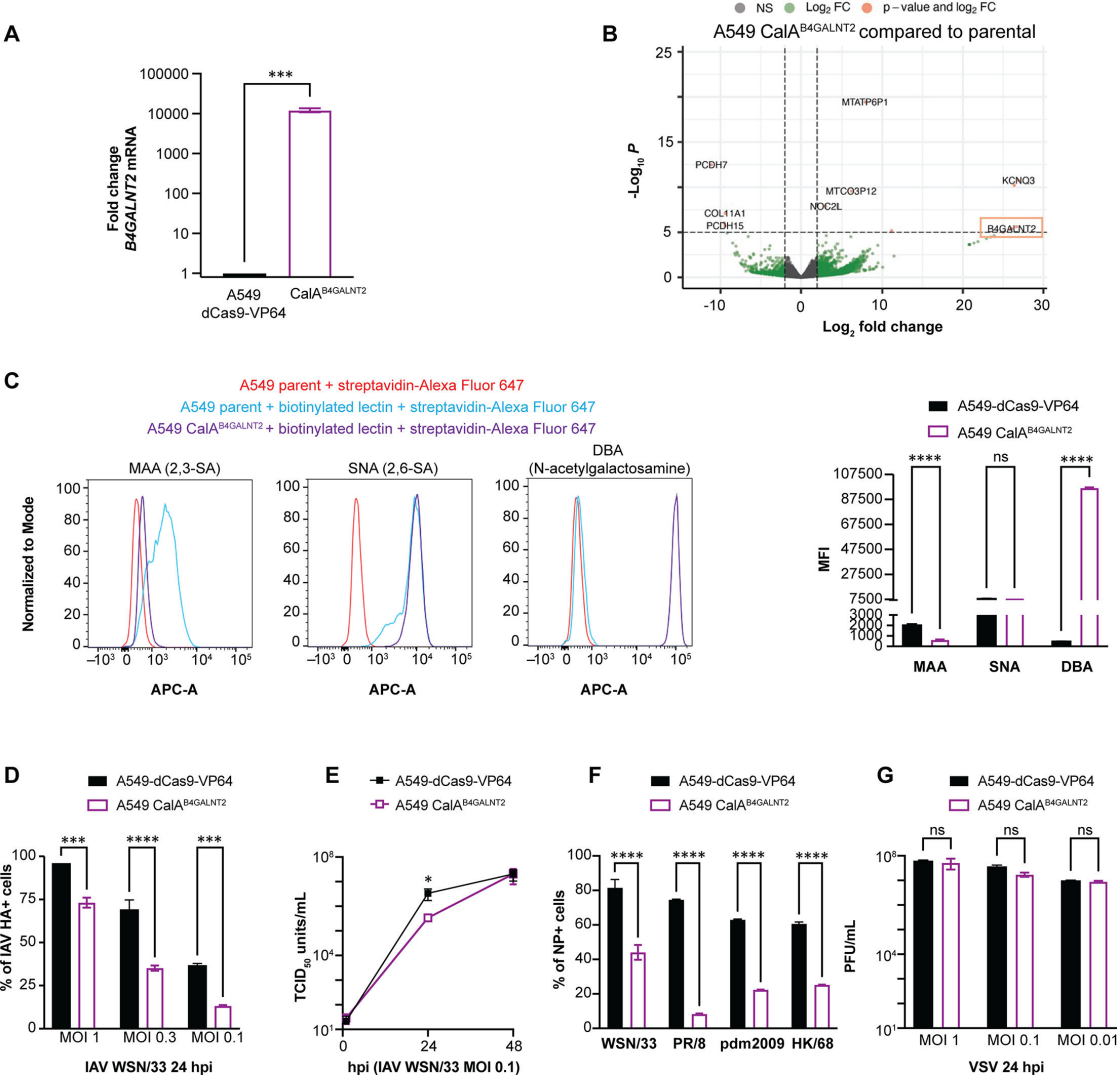
IAV infection is reduced, whereas VSV infection is sustained in A549 CalA^B4GALNT2^ cells. (A) Expression of *B4GALNT2* is increased in clonal A549 CalA^B4GALNT2^ cells compared with A549-dCas9-VP64 parent cells by RT-qPCR. RNA was extracted from each cell line and reverse transcribed. *B4GALNT2* and *HPRT* (housekeeping gene) were amplified, and the *B4GALNT2* fold change was calculated based on the 2^ΔΔCT^ method. ***, *P* < 0.001, unpaired *t*-test. (**B)** *B4GALNT2* expression is increased with few off-target effects in A549 CalA^B4GALNT2^ cells based on RNA-sequencing and differential expression analysis. Green dots indicate transcripts with log_2_FC > 2 or <-2 and −log_10_*P* > 5.** (C)** Flow cytometry histograms of binding of biotinylated MAA lectin (specific to α2,3-linked sialic acid), biotinylated SNA lectin (specific to α2,6-linked sialic acid), biotinylated DBA lectin (specific to N-acetylgalactosamine), and streptavidin-Alexa Fluor 647 to A549-dCas9-VP64 and A549 CalA^B4GALNT2^ cell lines. Each group has triplicate samples, and a representative sample from each group is shown. Quantification of mean fluorescence intensity (MFI) is shown. ****, *P* < 0.0001; ns = not significant, one-way ANOVA.** (D)** A549-dCas9-VP64 parent and CalA^B4GALNT2^ cells were infected with IAV WSN/33 at the indicated MOI. At 24 hpi, the percentage of HA+ cells was determined by anti-HA staining normalized to DAPI staining. ***, *P* < 0.001; ****, *P* < 0.0001, two-way ANOVA.** (E)** A549-dCas9-VP64 parent and CalA^B4GALNT2^ cells were infected with IAV WSN/33 at the indicated MOI. Supernatants were collected at 1, 24, and 48 hpi, and viral titers were measured by TCID_50_. *, *P* < 0.05, unpaired *t*-test.** (F)** A549-dCas9-VP64 parent and CalA^B4GALNT2^ cells were infected with IAV WSN/33, PR/8, pdm2009 (H1N1), and HK/68 (H3N2) using an inoculum at which ≥ 40% of A549-dCas9-VP64 cells are NP+ at 24 hpi (normalized to DAPI). ****, *P* < 0.0001, two-way ANOVA.** (G)** A549-dCas9-VP64 and CalA^B4GALNT2^ cells were infected with VSV at the indicated MOI. Supernatants were collected at 24 hpi, and PFU/mL was determined. ns = not significant, two-way ANOVA. Error bars represent the S.D. for triplicate samples.

A previous report showed that *B4GALNT2* overexpression preferentially impacts α2,3-sialic acid binding by adding N-acetylgalactosamine onto α2,3-sialic acid only and resulted in profound effects on infection with IAV strain PR/8 in A549 cells (29). In contrast, Wong et al. reported that MDCK cells overexpressing B4GALNT2 were equally permissive to IAV WSN/33 compared with parent MDCK cells (49). We were interested in following up on these findings, therefore transduced A549-dCas9-VP64 cells with lentivirus expressing B4GALNT2-specific sgRNA and established a clonal B4GALNT2-overexpressing cell line (CalA^B4GALNT2^) by limiting dilution. We confirmed overexpression of *B4GALNT2* by RT-qPCR and RNA-sequencing (RNA-seq) (Fig. 2A and B). Differential expression analysis revealed 15 additional transcripts with significantly increased expression in these cells (Table 1).

**TABLE 1:** RNA sequencing differential expression for A549 CalA^B4GALNT2^ cells compared with parent cells, log_2_ fold change >2.5

	Base mean	Log2 fold change	P value	Padj
*KCNQ3*	17.61	26.91	1.77E-11	1.18E-07
*NTRK2*	78.28	26.66	2.37E-06	4.74E-03
*PAK3*	16.55	26.38	6.24E-11	3.12E-07
*B4GALNT2*	910.94	26.07	3.93E-06	7.10E-03
*HS6ST2*	111.19	25.98	4.26E-06	7.10E-03
*JAKMIP2*	30.13	24.99	9.76E-06	1.39E-02
*NELL2*	37.13	23.88	2.38E-05	2.80E-02
*AFF3*	26.10	23.46	3.30E-05	3.30E-02
*MAPK4*	114.98	22.93	4.93E-05	4.70E-02
*NFATC4*	73.82	21.88	1.08E-04	9.81E-02
*VEPH1*	215.74	11.15	6.56E-06	1.01E-02
*MTATP6P1*	14,305.74	7.86	3.71E-20	7.43E-16
*MTCO3P12*	1,562.82	6.17	2.70E-10	1.08E-06
*EPHA5*	551.21	6.08	3.13E-05	3.30E-02
*SELENOP*	410.66	3.13	2.34E-05	2.80E-02
*NOC2L*	1,256.56	3.07	1.33E-08	4.44E-05

We tested for the effect of B4GALNT2 overexpression on sialic acid by performing lectin staining and flow cytometry as above. As expected, A549 CalA^B4GALNT2^ cells had less MAA (α2,3-sialic acid-specific) binding, no changes in SNA (α2,6-sialic acid-specific) binding, and increased binding to the N-acetylgalactosamine binding lectin *Dolichos biflorus* agglutinin (DBA) compared with parent cells (Fig. 2C). Infection of viruses with α2,3-receptor binding capability is expected to be diminished in cells with strong binding to DBA, a lectin that specifically binds to β−1,4 linked GalNAc residues (49).

Next, A549 CalA^B4GALNT2^ cells were challenged with IAV WSN/33 (H1N1) at the indicated MOI and fixed and stained with an anti-HA antibody at 24 hpi. IAV WSN/33 infection was modestly but significantly reduced in CalA^B4GALNT2^ cells compared with parent A549 cells by HA staining at 24 hpi and by growth assays performed at 24 hpi (Fig. 2D and E). The cells were then challenged with IAV strains PR/8 (H1N1), pdm2009 (H1N1), and HK/68 (H3N2) (Fig. 2F) to determine if there was a strain-specific effect. The greatest impairment in IAV infection was seen with the PR/8 strain passaged in embryonated chicken eggs, which is consistent with the findings by Heaton et al. (29). The loss of infectivity observed with CMAS deficiency was specific to IAV, as the titers of VSV recovered from the supernatant were similar between these cells and parent cells at 24 h following challenge with VSV at the indicated MOI (Fig. 2G).

### Loss of CMAS or overexpression of B4GALNT2 prevents binding and entry of IAV

**Fig 3:**
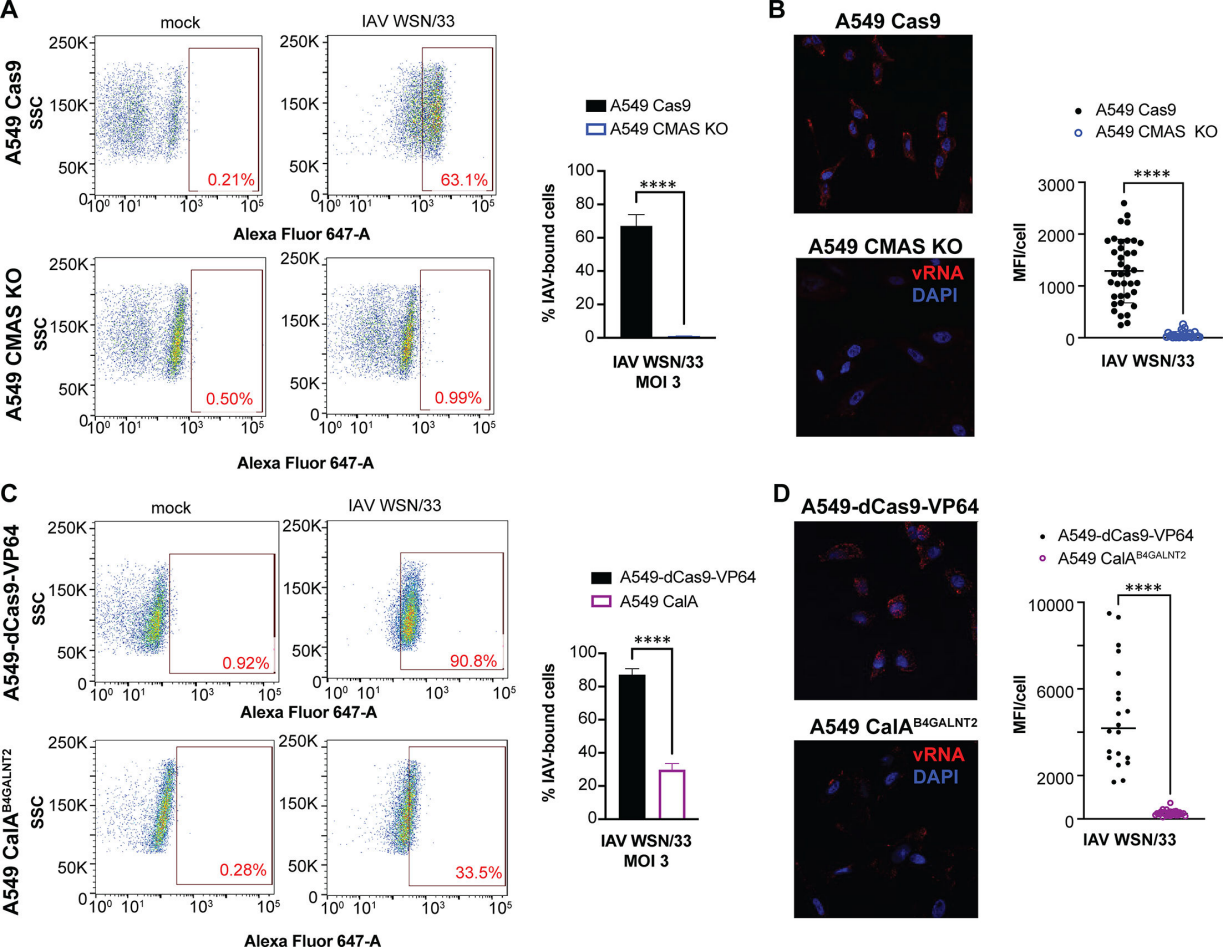
IAV binding and entry are decreased in A549 CMAS KO cells and A549 CalA^B4GALNT2^ cells compared with parent A549 cells. (A) Representative flow cytometry plots with quantification of IAV-bound A549 Cas9 parent and A549 CMAS KO cells. Cells were challenged with IAV WSN/33 at the indicated MOI (or mock-infected) for 1 h at 4°C to facilitate binding but not allow for endocytosis. Cells were washed, fixed, and then stained with anti-HA monoclonal antibody, followed by Alexa fluor 647-conjugated secondary antibody. IAV-bound cells were identified based on a comparison to anti-HA staining of mock-infected cells. The percentage of IAV^+^ cells is shown in red. All data were analyzed with FlowJo software. Quantification of the percentage of IAV-bound A549 Cas9 parent and A549 CMAS KO cells at the indicated MOI is shown. Error bars represent the S.D. for triplicate samples. ****, *P* < 0.0001, two-way ANOVA. (**B)** Representative composite immunofluorescence images were taken at 630× magnification for A549 Cas9 parent and A549 CMAS KO cells challenged with IAV WSN/33 at MOI 3 and fixed 20 min post-infection. IAV entry was defined by WSN/33 segment 7 vRNA staining by FISH (red), and cell nuclei were defined by DAPI staining (blue). The average mean fluorescence intensity (MFI, representing the average intensity of red staining) per cell was quantified and is shown in the graph. Each dot represents one cell, and the horizontal bar shows the mean value. Three images with at least 8 cells/field of view were captured for each cell line. ****, *P* < 0.0001, Mann-Whitney test.** (C)** Representative flow cytometry plots with quantification of IAV WSN/33-infected A549-dCas9-VP64 parent and A549 CalA^B4GALNT2^ cells. The approach was similar to that shown in A. (**D)** Representative composite immunofluorescence images taken at 630× magnification for A549-dCas9-VP64 parent and A549 CalA^B4GALNT2^ cells. The approach was similar to that for B.

We assessed whether the decrease in IAV infectivity of CMAS KO A549 cells is due to the lack of virus binding. We incubated prechilled parent and CMAS-deficient A549 cells with IAV WSN/33 on ice to facilitate virus binding but not entry. We observed a significant decrease in IAV binding at multiple MOIs tested in CMAS KO cells compared with parent A549 cells (Fig. 3). Entry was also restricted, as the fluorescence intensity of IAV WSN/33 vRNA post-entry was significantly reduced in CMAS-deficient cells compared with parent A549 cells as assayed by RNA FISH. Similarly, we tested for IAV WSN/33 binding and entry in A549 CalA^B4GALNT2^ cells by performing virus binding assays. Overexpression of B4GALNT2 resulted in interference with IAV WSN/33 infection through virus binding and internalization, although to a lesser degree than CMAS-deficient cells. Together, these results confirmed that altered expression of these host factors involved in sialic acid biosynthesis restricts IAV infection at the binding and entry stages.

### CRISPRa-mediated overexpression of ADAR1 p150 decreases IAV infectivity

ADAR1 was of interest, given that both pro-viral and anti-viral roles have been ascribed to this factor, and the consequences of overexpressing the ADAR1 p150 isoform during IAV infection have not been explored. Because the sgRNAs in the library were designed to target the p150 promoter, we elected to overexpress ADAR1 in A549 cells using gRNA specific for the p150 promoter to define its impact on infection by IAV and other viruses.

**Fig 4:**
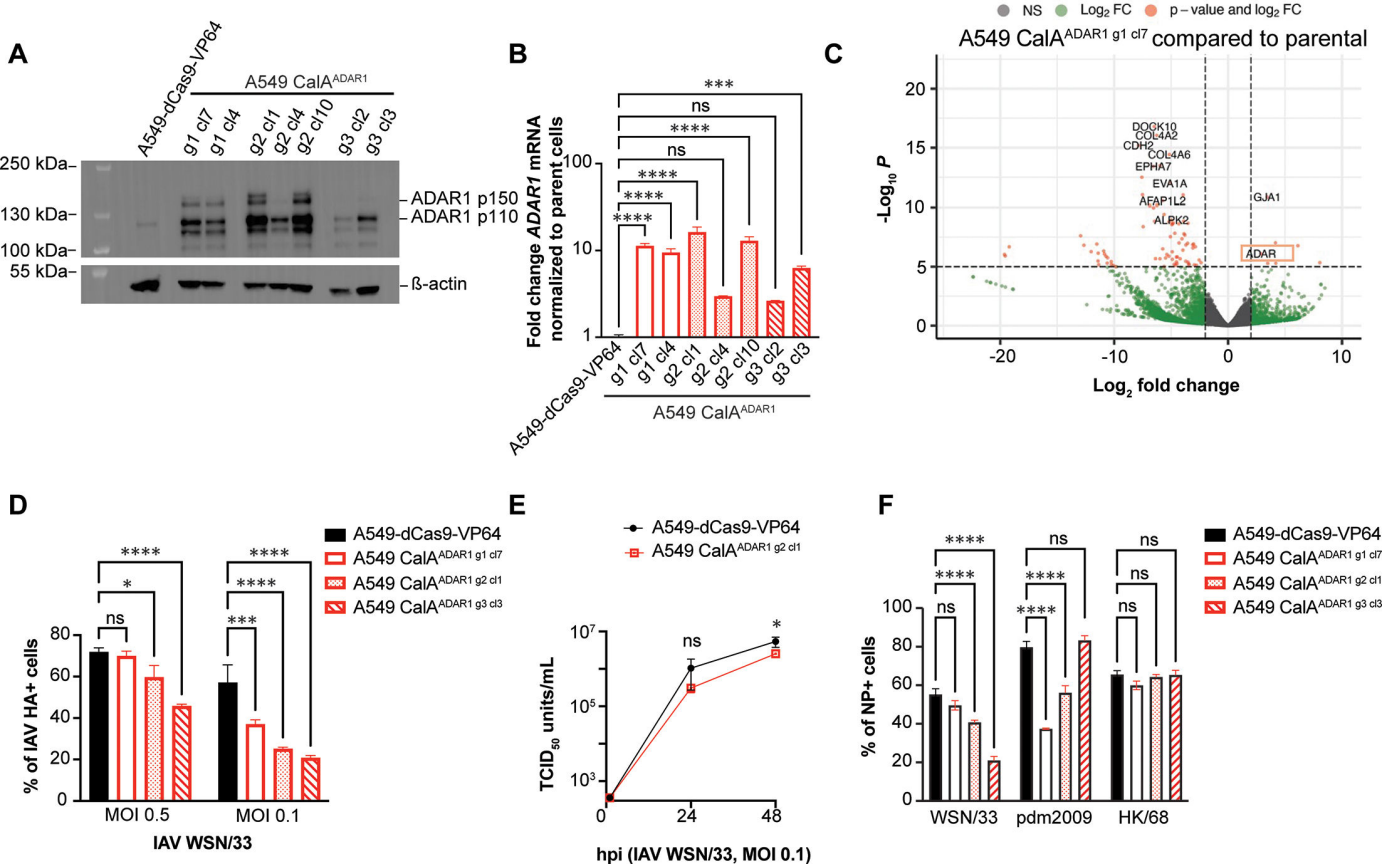
ADAR1 overexpression in A549 CalA^ADAR1^ clonal cells is associated with a modest reduction in IAV infection. (A) Equal protein concentrations of CalA^ADAR1^ clonal cells and A549-dCas9-VP64 parent cells were loaded onto a 4%–8% Tris-Acetate gel for SDS-PAGE electrophoresis and transferred to a PVDF membrane, which was then probed with antibodies against ADAR1 and actin. Chemiluminescent substrate was added, and the blot was imaged. (**B)** Expression of *ADAR1* expression in clonal A549 CalA^ADAR1^ cells is increased compared with A549-dCas9-VP64 parent cells by RT-qPCR. RNA was extracted from each cell line and reverse transcribed. *ADAR1* and *HPRT* (housekeeping gene) were amplified, and the *ADAR1* fold change was calculated based on the 2^ΔΔCT^ method. ***, *P* < 0.001; ****, *P* < 0.0001; ns = not significant, one-way ANOVA. Error bars represent the S.D. for triplicate samples. The primers for ADAR1 bind to exon 2 which is expressed in both p110 and p150 isoforms. (**C)** *ADAR1* expression is increased with some off-target effects in A549 CalA^ADAR1 g1 cl7^ cells based on RNA-sequencing and differential expression analysis. Transcripts with log_2_FC > 2 or <-2 are green and those with −log_10_*P* > 5 are orange.** (D)** A549-dCas9-VP64 parent and A549 CalA^ADAR1^ clonal cells were infected with IAV WSN/33 at the indicated MOI. At 24 hpi, the percentage of HA+ cells was determined by anti-HA staining normalized to DAPI staining. *, *P* < 0.05; ***, *P* < 0.001; ****, *P* < 0.0001; ns = not significant by two-way ANOVA.** (E)** A549-dCas9-VP64 parent and A549 CalA^ADAR1 g2 cl1^ cells were infected with IAV WSN/33 at the indicated MOI. Supernatants were collected at 1, 24, and 48 hpi, and viral titers were measured by TCID_50_. *, *P* < 0.05; ns = not significant by unpaired t test.** (F)** A549-dCas9-VP64 parent and CalA^ADAR1^ cells were infected with IAV WSN/33, pdm2009 (H1N1), and HK/68 (H3N2) using an inoculum at which ≥ 40% of A549-dCas9-VP64 cells are NP+ at 24 hpi (normalized to DAPI). ***, *P* < 0.001; ****, *P* < 0.0001; ns = not significant, two-way ANOVA. Error bars represent the S.D. for triplicate samples.

We transduced three different sgRNAs targeting the ADAR1 p150 promoter into A549-dCas9-VP64 cells (see Materials and Methods) and established clonal cell lines (CalA^ADAR1^) post-selection by limiting dilution. By western blot, we showed that ADAR1 p150 was overexpressed in clonal CalA^ADAR1^ cell lines with guide 1 (clones 7 and 4) and guide 2 (clones 1 and 10). Overexpression of the p110 isoform is observed in all CalA^ADAR1^ clonal cell lines by western blot, which may result from leaky ribosome scanning during transcription (50). Upregulation of *ADAR1* was observed in all clones by RT-qPCR using non-isoform-specific primers. We also detected upregulation of *ADAR1* by next-generation sequencing of CalA^ADAR1 g1 cl7^ cells. Off-target effects in these cells were more prevalent than for *B4GALNT2* overexpressing cells (Fig. 4C; Table 2). Given the potential for off-target effects, we examined three different A549 CalA^ADAR1^ clones (A549 CalA^ADAR1 g1 cl7^, A549 CalA^ADAR1 g2 cl1^, and A549 CalA^ADAR1 g3 cl3^) in our IAV infection studies.

**TABLE 2:** RNA-sequencing differential expression for A549 CalA^ADAR1^ cells compared to parent cells, log_2_ fold change >2.5

	Base mean	Log2 fold change	P value	Padj
*ZNF700*	15.84	8.50	6.48E-04	5.06E-02
*ZNF136*	16.18	8.16	3.10E-04	2.97E-02
*OTOF*	15.85	8.16	3.41E-04	3.19E-02
*COL11A1*	41.63	8.06	4.57E-06	1.07E-03
*ZNF91*	72.03	6.13	1.68E-07	6.97E-05
*ZSCAN18*	36.83	5.60	1.03E-03	7.03E-02
*PCDH7*	100.48	5.44	5.61E-04	4.67E-02
*CHGB*	8,093.17	5.28	4.65E-05	6.59E-03
*CALCRL*	126.78	5.03	6.18E-04	4.91E-02
*GUCY1A2*	70.09	4.87	2.25E-04	2.25E-02
*SBK2*	156.16	4.83	3.60E-04	3.33E-02
*ZNF420*	33.45	4.83	4.58E-04	4.04E-02
*IGSF10*	478.06	4.65	1.64E-03	9.87E-02
*SERPINI1*	760.60	4.19	5.01E-06	1.14E-03
*COL26A1*	135.37	4.16	9.57E-08	4.90E-05
*PTCH1*	1,146.09	3.78	1.81E-05	3.04E-03
*SNTB1*	326.84	3.54	2.99E-05	4.57E-03
*BDKRB1*	87.98	3.51	1.15E-03	7.78E-02
*DLK2*	102.74	3.48	5.13E-06	1.15E-03
*GJA1*	8,959.22	3.43	1.52E-11	2.35E-08
*PDE3B*	734.23	3.39	1.35E-04	1.51E-02
*HOXC8*	172.02	3.22	4.11E-04	3.72E-02
*PCDH1*	159.87	3.17	4.95E-04	4.23E-02
*PARM1*	499.23	2.97	2.23E-05	3.65E-03
*CA11*	549.82	2.90	5.03E-04	4.26E-02
*ADAR*	107,586.14	2.88	7.84E-07	2.61E-04
*HOXB8*	307.58	2.55	3.30E-04	3.12E-02
*COL12A1*	1,978.50	2.53	1.27E-03	8.34E-02
*RAP1GAP2*	2,318.42	2.51	8.48E-04	6.11E-02

Three CalA^ADAR1^ clonal cell lines and parent A549-dCas9-VP64 cells were challenged with IAV WSN/33 at two different MOI, and then, 24 hours later, they were fixed and stained for HA to determine the percentage of infection. Interestingly, IAV WSN/33 was significantly but only modestly inhibited as determined by HA staining of these various A549 CalA^ADAR1^ cell lines (Fig. 4D). Moreover, a viral growth assay quantifying the IAV WSN/33 TCID_50_ over 48 h showed a reduction in titers of less than 10-fold for A549 CalA^ADAR1 g2 cl1^ cells compared with parent cells (Fig. 4E), and trends with IAV pdm2009 and HK/68 strains were inconsistent with those for WSN/33 (Fig. 4F). Altogether, these data do not support a strong anti-IAV role for ADAR1, either p110 or p150, in A549 cells.

### Overexpression of ADAR1 results in increased infection of CVB but not VSV

**Fig 5:**
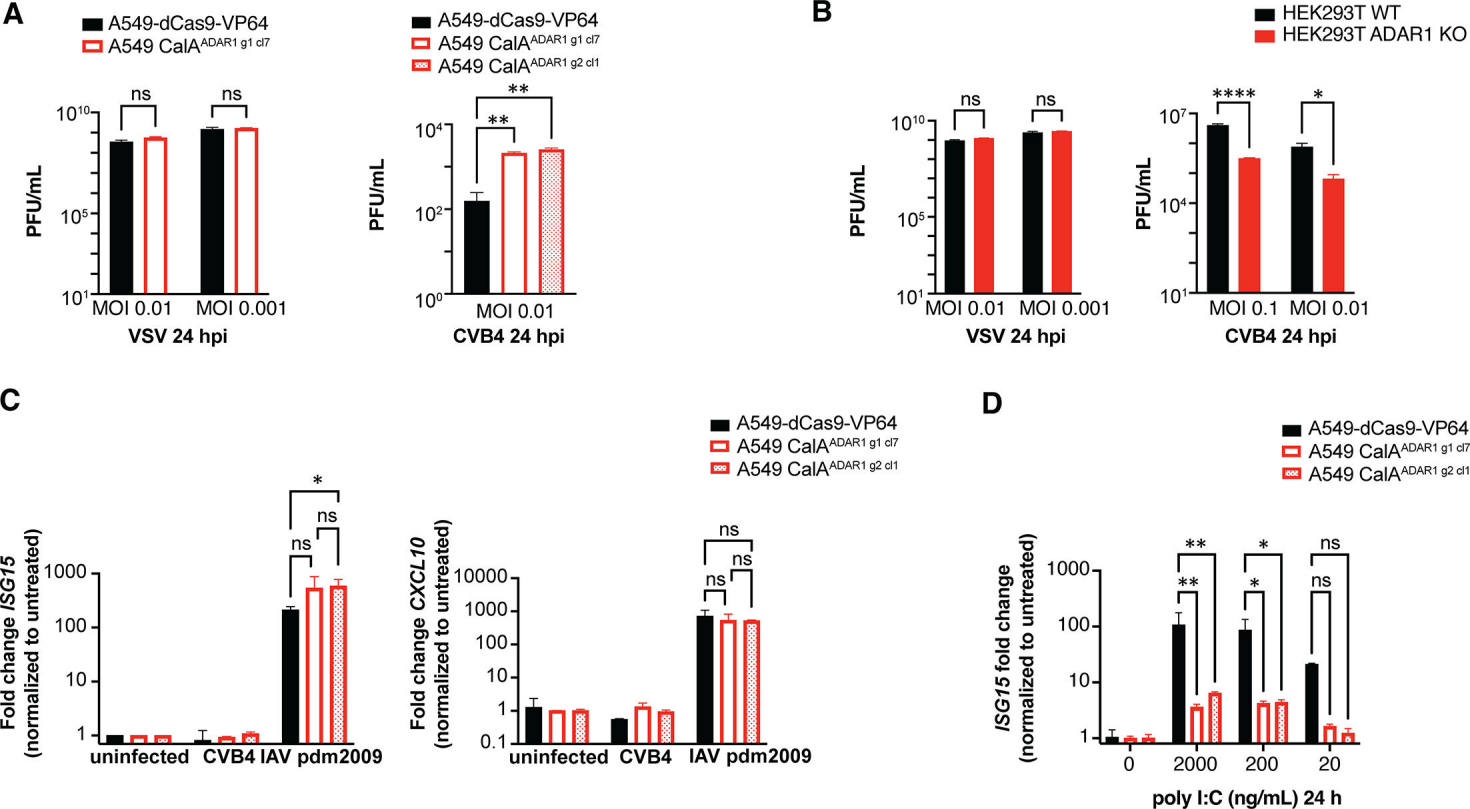
CVB4 replication is increased in A549 CalA^ADAR1^ clonal cells, and the interferon-stimulated gene response is absent in response to CVB infection in both A549 parent and A549 CalA^ADAR1^ clonal cells. (A) A549-dCas9-V64 parent and A549 CalA^ADAR1^ cells were infected with VSV or CVB4 at the indicated MOI. At 24 hpi, the supernatants were collected and titered. ns = not significant by one-way ANOVA for VSV. **, *P* < 0.01 by one-way ANOVA for CVB4.** (B)** HEK293T parent and ADAR1 KO cells were infected with VSV or CVB4 at the indicated MOI. At 24 hpi, supernatants were collected and titered. Error bars represent the S.D. for triplicate samples. ns = not significant for VSV. ****, *P* < 0.0001; *, *P* < 0.05 by one-way ANOVA for CVB4.** (C)** A549-dCas9-VP64 parent and A549 CalA^ADAR1^ cells were infected with CVB4 at MOI 0.1 or IAV pdm2009 at MOI 0.3 for 24 h. RNA was extracted from each cell line and reverse transcribed. *ISG15, CXCL10,* and *HPRT* (housekeeping gene) were amplified, and the *ISG15* or *CXCL10* fold change was calculated based on the 2^ΔΔCT^ method. *, *P* < 0.05; ns = not significant, one-way ANOVA. Error bars represent the S.D. for triplicate samples. (**D)** A549-dCas9-VP64 parent and A549 CalA^ADAR1^ cells were treated with dsRNA mimetic poly I:C at the indicated concentrations for 24 h. *ISG15* and *HPRT* (housekeeping gene) were amplified, and the *ISG15* fold change was calculated based on the 2^ΔΔCT^ method. **, *P* < 0.01; *, *P* < 0.05; ns = not significant, one-way ANOVA. Error bars represent the S.D. for triplicate samples.

To determine if ADAR1 overexpression impacts other viruses, the A549 CalA^ADAR1 g1 cl7^ clonal cell line was challenged with VSV at the indicated MOI for 24 h. Supernatants were collected and titered on BHK-21 cells. No difference in the VSV titers was observed from the supernatant of CalA^ADAR1^ cells compared with that from the parent A549-dCas9-VP64 cells (Fig. 5A**, left**). On the other hand, ADAR1 overexpression was robustly pro-viral during CVB4 infection, as a 10-fold increase in viral titers was observed in the supernatant from two different CalA^ADAR1^ cell lines compared with A549-dCas9-VP64 cells at 24 hpi (Fig. 5A**, right**). To test how ADAR1 deficiency impacts CVB replication, we challenged HEK293T WT and HEK293T ADAR1 KO cells lacking both the p110 and p150 isoforms (35) with CVB4 strain JVB. As expected, the absence of ADAR1 resulted in a significant decrease in CVB4 titers at 24 hpi but had no effect on VSV replication (Fig. 5B). Thus, our data support a pro-viral role for ADAR1 during CVB4 infection.

Next, we considered whether the pro-viral effect of *ADAR1* overexpression during CVB4 infection is linked to a diminished IFN response. Because IFN production following CVB infection is largely mediated by MDA5 (51), we hypothesized that ADAR1 overexpression diverts dsRNA replicative intermediates from engaging with MDA5. Thus, we challenged A549-dCas9-VP64 parent cells and A549 CalA^ADAR1^ cells with CVB4 and extracted RNA from the cells at 24 hpi to evaluate ISG expression using RT-qPCR. Unexpectedly, *ISG15* and *CXCL10* are not induced following CVB4 infection of either CalA^ADAR1^ or A549-dCas9-VP64 cells (Fig. 5C); this result was robust and consistent but surprising, given that CVB4 induces ISGs in other cell systems such as human islets (52, 53). To exclude a generalized defect in ISG production, A549-dCas9-VP64 parent and CalA^ADAR1^ cells were infected with IAV pdm2009, and RNA was extracted at 24 hpi for RT-qPCR. *ISG15* and *CXCL10* are highly and equally induced in A549 CalA^ADAR1^ and A549-dCas9-VP64 cells (Fig. 5C), confirming that the ISG response in A549 CalA^ADAR1^ cells is intact. We also treated cells with the dsRNA mimetic poly I:C to induce an ISG response. At 24 h post-treatment, *ISG15* induction is present but significantly dampened in CalA^ADAR1^ cells compared with A549-dCas9-VP64 cells (Fig. 5D). Here, poly I:C may be sequestered by ADAR1 and diverted away from detection by MDA5. Further studies in diverse host cells are needed to determine whether such findings are specific to A549 cells or can be observed more broadly.

## DISCUSSION

In this study, we demonstrate that the roles of host genes involved in productive virus infection can be identified either through either knockout, as exemplified by *CMAS*, or through overexpression, as for *B4GALNT2* and *ADAR1*. CMAS is an essential enzyme in the sialic acid biosynthesis pathway catalyzing the rate-limiting step of converting N-acetylneuraminic acid to 5-monophosphate N-acetylneuraminic acid in the nucleus, which is an essential building block for sialic acid (54). CRISPR-Cas9-mediated deletion of *CMAS* disrupts sialic acid biosynthesis, resulting in minimal production of α2,3- and α2,6-linked sialic acids on the cell surface. Consequently, we demonstrate that CMAS KO cells are less permissive to IAV infection due to impaired virus binding and that sialidase treatment eliminates any residual infection.

*B4GALNT2* is responsible for the addition of N-acetylgalactosamine onto the subterminal galactose of the α2,3-linked sialic acid monosaccharide. *B4GALNT2* has been reported to influence IAV (29). We observed varying levels of resistance to infection by different IAV strains in CalA^B4GALNT2^ cells, perhaps due to the preference of each strain for either α2,3- or α2,6-linked sialic acid. Since overexpression of *B4GALNT2* specifically modifies α2,3-linked sialic acid, CalA^B4GALNT2^ cells can be used to distinguish the dependency of different viruses on either α2,3- or α2,6-linked sialic acids.

ADAR1 catalyzes the deamination of adenosine to inosine (A-to-I) on dsRNA, thereby preventing aberrant IFN secretion in the absence of virus infection by masking endogenous dsRNA from detection by RLRs. ADAR1 can function as either a pro- or anti-viral host factor depending on the virus (32). In the context of IAV infection, ADAR1 p150 has been reported to be a pro-viral host factor functioning to block RLR signaling and apoptosis, and ADAR1 p110 has been shown to be an anti-viral host factor (34). ADAR1 can also act as an anti-viral host factor through A-to-I editing of matrix (M) mRNA (55). However, specific overexpression of ADAR1 in A549 cells had very little inhibitory effect during IAV WSN/33 infection. A549 cells containing guide 3, which appeared to have increased levels of p110 but not p150 on western blot (Fig. 4A), had a greater reduction of IAV WSN/33 infection compared with those with guide 1 or 2. This is consistent with the report by Vogel et al. that ADAR1 p110 expression hinders IAV replication.

Interestingly, we found that ADAR1 overexpression had a pro-viral effect during infection with CVB4. Two prototypical ISGs, *ISG15* and *CXCL10,* are not induced in parent or ADAR1-overexpressing A549 cells following CVB4 challenge. IFN signaling and ISG induction are intact in both parent and ADAR1-overexpressing cells, as *ISG15* and *CXCL10* are induced robustly and equally following IAV challenge. However, reduced expression of ISGs in poly I:C-challenged ADAR1-overexpressing A549 cells may be due to ADAR1 diverting poly I:C away from downstream interactions with MDA5, but this did not appear to be the case for CVB4. Pro-viral effects of ADAR1 overexpression during CVB4 infection could be due to ADAR1 interactions with protein kinase R (PKR), as ADAR1 p150 suppresses PKR activation by competitive binding to dsRNA (51, 52), whereas a functional PKR is a classical cytosolic dsRNA sensor that shuts off viral protein synthesis and reduces virus replication (56). Since both ADAR1 p150 and ADAR1 p110 are induced in our CRISPRa-modified cells, despite our design to specifically upregulate the p150 isoform, future studies should express these isoforms from cDNAs to decouple the contribution of each in the context of CVB4 and IAV infection. Additionally, the blockade of PKR could be used to confirm its possible role in ADAR1-driven pro-CVB4 effects.

In sum, our work demonstrates that CRISPR-based modulation strategies can identify host factors that could serve as novel targets for antiviral strategies against IAV. The assays we developed help elucidate the role of such host factors during the infection process. Furthermore, they provide insights into how these factors contribute to infection with other viruses.

## References

[1] Fineberg HV. 2014. Pandemic preparedness and response--lessons from the H1N1 influenza of 2009. N Engl J Med 370:1335–1342. doi:10.1056/NEJMra1208802 24693893

[2] Hao L, Sakurai A, Watanabe T, Sorensen E, Nidom CA, Newton MA, Ahlquist P, Kawaoka Y. 2008. Drosophila RNAi screen identifies host genes important for influenza virus replication. Nature 454:890–893. doi:10.1038/nature07151 18615016 PMC2574945

[3] Brass AL, Huang I-C, Benita Y, John SP, Krishnan MN, Feeley EM, Ryan BJ, Weyer JL, van der Weyden L, Fikrig E, Adams DJ, Xavier RJ, Farzan M, Elledge SJ. 2009. The IFITM proteins mediate cellular resistance to influenza A H1N1 virus, West Nile virus, and dengue virus. Cell 139:1243–1254. doi:10.1016/j.cell.2009.12.017 20064371 PMC2824905

[4] Shapira SD, Gat-Viks I, Shum BOV, Dricot A, de Grace MM, Wu L, Gupta PB, Hao T, Silver SJ, Root DE, Hill DE, Regev A, Hacohen N. 2009. A physical and regulatory map of host-influenza interactions reveals pathways in H1N1 infection. Cell 139:1255–1267. doi:10.1016/j.cell.2009.12.018 20064372 PMC2892837

[5] König R, Stertz S, Zhou Y, Inoue A, Hoffmann H-H, Bhattacharyya S, Alamares JG, Tscherne DM, Ortigoza MB, Liang Y. 2010. Human host factors required for influenza virus replication. Nature 463:813–817. doi:10.1038/nature08699 20027183 PMC2862546

[6] Karlas A, Machuy N, Shin Y, Pleissner K-P, Artarini A, Heuer D, Becker D, Khalil H, Ogilvie LA, Hess S, Mäurer AP, Müller E, Wolff T, Rudel T, Meyer TF. 2010. Genome-wide RNAi screen identifies human host factors crucial for influenza virus replication. Nature 463:818–822. doi:10.1038/nature08760 20081832

[7] Su W-C, Chen Y-C, Tseng C-H, Hsu PW-C, Tung K-F, Jeng K-S, Lai MMC. 2013. Pooled RNAi screen identifies ubiquitin ligase Itch as crucial for influenza a virus release from the endosome during virus entry. Proc Natl Acad Sci U S A 110:17516–17521. doi:10.1073/pnas.1312374110 24101521 PMC3808593

[8] Chou YC, Lai MM, Wu YC, Hsu NC, Jeng KS, Su WC. 2015. Variations in genome-wide RNAi screens: lessons from influenza research. J Clin Bioinforma 5:2. doi:10.1186/s13336-015-0017-5 25745555 PMC4350949

[9] Cong L, Ran FA, Cox D, Lin S, Barretto R, Habib N, Hsu PD, Wu X, Jiang W, Marraffini LA, Zhang F. 2013. Multiplex genome engineering using CRISPR/Cas systems. Science 339:819–823. doi:10.1126/science.1231143 23287718 PMC3795411

[10] Jinek M, East A, Cheng A, Lin S, Ma E, Doudna J. 2013. RNA-programmed genome editing in human cells. Elife 2:e00471. doi:10.7554/eLife.00471 23386978 PMC3557905

[11] Mali P, Yang L, Esvelt KM, Aach J, Guell M, DiCarlo JE, Norville JE, Church GM. 2013. RNA-guided human genome engineering via Cas9. Science 339:823–826. doi:10.1126/science.1232033 23287722 PMC3712628

[12] Li B, Clohisey SM, Chia BS, Wang B, Cui A, Eisenhaure T, Schweitzer LD, Hoover P, Parkinson NJ, Nachshon A, Smith N, Regan T, Farr D, Gutmann MU, Bukhari SI, Law A, Sangesland M, Gat-Viks I, Digard P, Vasudevan S, Lingwood D, Dockrell DH, Doench JG, Baillie JK, Hacohen N. 2020. Genome-wide CRISPR screen identifies host dependency factors for influenza a virus infection. Nat Commun 11:164. doi:10.1038/s41467-019-13965-x 31919360 PMC6952391

[13] Han J, Perez JT, Chen C, Li Y, Benitez A, Kandasamy M, Lee Y, Andrade J, tenOever B, Manicassamy B. 2018. Genome-wide CRISPR/Cas9 screen identifies host factors essential for influenza virus replication. Cell Rep 23:596–607. doi:10.1016/j.celrep.2018.03.045 29642015 PMC5939577

[14] Ma T, Niu S, Wu Z, Pan S, Wang C, Shi X, Yan M, Xu B, Liu X, Li L, Yan D, Teng Q, Yuan C, Pan X, Zhang Z, Duc HM, Li Z, Liu Q. 2023. Genome-wide CRISPR screen identifies GNE as A key host factor that promotes influenza a virus adsorption and endocytosis. Microbiol Spectr 11:e01643–23. doi:10.1128/spectrum.01643-23

[15] Wei J, Alfajaro MM, DeWeirdt PC, Hanna RE, Lu-Culligan WJ, Cai WL, Strine MS, Zhang S-M, Graziano VR, Schmitz CO. 2021. Genome-wide CRISPR screens reveal host factors critical for SARS-CoV-2 infection. Cell 184:76–91. doi:10.1016/j.cell.2020.10.028 33147444 PMC7574718

[16] Daniloski Z, Jordan TX, Wessels H-H, Hoagland DA, Kasela S, Legut M, Maniatis S, Mimitou EP, Lu L, Geller E, Danziger O, Rosenberg BR, Phatnani H, Smibert P, Lappalainen T, tenOever BR, Sanjana NE. 2021. Identification of required host factors for SARS-CoV-2 infection in human cells. Cell 184:92–105. doi:10.1016/j.cell.2020.10.030 33147445 PMC7584921

[17] Wang R, Simoneau CR, Kulsuptrakul J, Bouhaddou M, Travisano KA, Hayashi JM, Carlson-Stevermer J, Zengel JR, Richards CM, Fozouni P, Oki J, Rodriguez L, Joehnk B, Walcott K, Holden K, Sil A, Carette JE, Krogan NJ, Ott M, Puschnik AS. 2021. Genetic screens identify host factors for SARS-CoV-2 and common cold coronaviruses. Cell 184:106–119. doi:10.1016/j.cell.2020.12.004 33333024 PMC7723770

[18] Lin DL, Cherepanova NA, Bozzacco L, MacDonald MR, Gilmore R, Tai AW. 2017. Dengue virus hijacks a noncanonical oxidoreductase function of a Cellular oligosaccharyltransferase complex. MBio 8:e00939-17. doi:10.1128/mBio.00939-17 28720733 PMC5516256

[19] Hoffmann HH, Schneider WM, Rozen-Gagnon K, Miles LA, Schuster F, Razooky B, Jacobson E, Wu X, Yi S, Rudin CM, MacDonald MR, McMullan LK, Poirier JT, Rice CM. 2021. TMEM41B Is a pan-flavivirus host factor. Cell 184:133–148. doi:10.1016/j.cell.2020.12.005 33338421 PMC7954666

[20] Wang S, Zhang Q, Tiwari SK, Lichinchi G, Yau EH, Hui H, Li W, Furnari F, Rana TM. 2020. Integrin αvβ5 internalizes zika virus during neural stem cells infection and provides a promising target for antiviral therapy. Cell Rep 30:969–983. doi:10.1016/j.celrep.2019.11.020 31956073 PMC7293422

[21] Yang X, Wang Y, Lu P, Shen Y, Zhao X, Zhu Y, Jiang Z, Yang H, Pan H, Zhao L, Zhong Y, Wang J, Liang Z, Shen X, Lu D, Jiang S, Xu J, Wu H, Lu H, Jiang G, Zhu H. 2020. PEBP1 suppresses HIV transcription and induces latency by inactivating MAPK/NF-κB signaling. EMBO Rep 21:e49305. doi:10.15252/embr.201949305 32924251 PMC7645261

[22] Krasnopolsky S, Kuzmina A, Taube R. 2020. Genome-wide CRISPR knockout screen identifies ZNF304 as a silencer of HIV transcription that promotes viral latency. PLoS Pathog 16:e1008834. doi:10.1371/journal.ppat.1008834 32956422 PMC7529202

[23] Rathore A, Iketani S, Wang P, Jia M, Sahi V, Ho DD. 2020. CRISPR-based gene knockout screens reveal deubiquitinases involved in HIV-1 latency in two Jurkat cell models. Sci Rep 10:5350. doi:10.1038/s41598-020-62375-3 32210344 PMC7093534

[24] Yi C, Cai C, Cheng Z, Zhao Y, Yang X, Wu Y, Wang X, Jin Z, Xiang Y, Jin M, Han L, Zhang A. 2022. Genome-wide CRISPR-Cas9 screening identifies the CYTH2 host gene as a potential therapeutic target of influenza viral infection. Cell Rep 38:110559. doi:10.1016/j.celrep.2022.110559 35354039

[25] Qi LS, Larson MH, Gilbert LA, Doudna JA, Weissman JS, Arkin AP, Lim WA. 2013. Repurposing CRISPR as an RNA-guided platform for sequence-specific control of gene expression. Cell 152:1173–1183. doi:10.1016/j.cell.2013.02.022 23452860 PMC3664290

[26] Gilbert LA, Larson MH, Morsut L, Liu Z, Brar GA, Torres SE, Stern-Ginossar N, Brandman O, Whitehead EH, Doudna JA, Lim WA, Weissman JS, Qi LS. 2013. CRISPR-mediated modular RNA-guided regulation of transcription in eukaryotes. Cell 154:442–451. doi:10.1016/j.cell.2013.06.044 23849981 PMC3770145

[27] Gilbert LA, Horlbeck MA, Adamson B, Villalta JE, Chen Y, Whitehead EH, Guimaraes C, Panning B, Ploegh HL, Bassik MC, Qi LS, Kampmann M, Weissman JS. 2014. Genome-scale CRISPR-mediated control of gene repression and activation. Cell 159:647–661. doi:10.1016/j.cell.2014.09.029 25307932 PMC4253859

[28] Konermann S, Brigham MD, Trevino AE, Joung J, Abudayyeh OO, Barcena C, Hsu PD, Habib N, Gootenberg JS, Nishimasu H, Nureki O, Zhang F. 2015. Genome-scale transcriptional activation by an engineered CRISPR-Cas9 complex. Nature 517:583–588. doi:10.1038/nature14136 25494202 PMC4420636

[29] Heaton BE, Kennedy EM, Dumm RE, Harding AT, Sacco MT, Sachs D, Heaton NS. 2017. A CRISPR activation screen identifies a pan-avian influenza virus inhibitory host factor. Cell Rep 20:1503–1512. doi:10.1016/j.celrep.2017.07.060 28813663 PMC5568676

[30] Song C, Sakurai M, Shiromoto Y, Nishikura K. 2016. Functions of the RNA editing enzyme ADAR1 and their relevance to human diseases. Genes (Basel) 7:129. doi:10.3390/genes7120129 27999332 PMC5192505

[31] Crow YJ, Chase DS, Lowenstein Schmidt J, Szynkiewicz M, Forte GMA, Gornall HL, Oojageer A, Anderson B, Pizzino A, Helman G, et al.. 2015. Characterization of human disease phenotypes associated with mutations in TREX1, RNASEH2A, RNASEH2B, RNASEH2C, SAMHD1, ADAR, and IFIH1. Am J Med Genet A 167A:296–312. doi:10.1002/ajmg.a.36887 25604658 PMC4382202

[32] Pfaller CK, George CX, Samuel CE. 2021. Adenosine deaminases acting on RNA (ADARs) and viral infections. Annu Rev Virol 8:239–264. doi:10.1146/annurev-virology-091919-065320 33882257 PMC12925331

[33] Ward SV, George CX, Welch MJ, Liou L-Y, Hahm B, Lewicki H, de la Torre JC, Samuel CE, Oldstone MB. 2011. RNA editing enzyme adenosine deaminase is a restriction factor for controlling measles virus replication that also is required for embryogenesis. Proc Natl Acad Sci U S A 108:331–336. doi:10.1073/pnas.1017241108 21173229 PMC3017198

[34] Vogel OA, Han J, Liang CY, Manicassamy S, Perez JT, Manicassamy B. 2020. The p150 isoform of ADAR1 blocks sustained RLR signaling and apoptosis during influenza virus infection. PLoS Pathog 16:e1008842. doi:10.1371/journal.ppat.1008842 32898178 PMC7500621

[35] Pestal K, Funk CC, Snyder JM, Price ND, Treuting PM, Stetson DB. 2015. Isoforms of RNA-editing enzyme ADAR1 independently control nucleic acid sensor MDA5-driven autoimmunity and multi-organ development. Immunity 43:933–944. 26588779 10.1016/j.immuni.2015.11.001 PMC4654992

[36] Chen Y, Lei X, Jiang Z, Fitzgerald KA. 2021. Cellular nucleic acid–binding protein is essential for type I interferon–mediated immunity to RNA virus infection. Proc Natl Acad Sci USA 118. doi:10.1073/pnas.2100383118 PMC8255963 34168080

[37] Sanson KR, Hanna RE, Hegde M, Donovan KF, Strand C, Sullender ME, Vaimberg EW, Goodale A, Root DE, Piccioni F, Doench JG. 2018. Optimized libraries for CRISPR-Cas9 genetic screens with multiple modalities. Nat Commun 9:5416. doi:10.1038/s41467-018-07901-8 30575746 PMC6303322

[38] Dobin A, Davis CA, Schlesinger F, Drenkow J, Zaleski C, Jha S, Batut P, Chaisson M, Gingeras TR. 2013. STAR: ultrafast universal RNA-seq aligner. Bioinformatics 29:15–21. doi:10.1093/bioinformatics/bts635 23104886 PMC3530905

[39] Li B, Dewey CN. 2011. RSEM: accurate transcript quantification from RNA-Seq data with or without a reference genome. BMC Bioinformatics 12:323. doi:10.1186/1471-2105-12-323 21816040 PMC3163565

[40] Yukselen O, Turkyilmaz O, Ozturk AR, Garber M, Kucukural A. 2020. DolphinNext: a distributed data processing platform for high throughput genomics. BMC Genomics 21:310. doi:10.1186/s12864-020-6714-x 32306927 PMC7168977

[41] Love MI, Huber W, Anders S. 2014. Moderated estimation of fold change and dispersion for RNA-seq data with DESeq2. Genome Biol 15:550. doi:10.1186/s13059-014-0550-8 25516281 PMC4302049

[42] Bligh K, Rana S, Lewis M. 2019. EnhancedVolcano: publication-ready volcano plots with enhanced colouring and labeling. R package version 1

[43] Balish AL, Katz JM, Klimov AI. 2013. Influenza: propagation, quantification, and storage. Curr Protoc Microbiology 29:1–15. doi:10.1002/9780471729259.mc15g01s29 23686827

[44] Prachanronarong KL, Canale AS, Liu P, Somasundaran M, Hou S, Poh YP, Han T, Zhu Q, Renzette N, Zeldovich KB, Kowalik TF, Kurt-Yilmaz N, Jensen JD, Bolon DNA, Marasco WA, Finberg RW, Schiffer CA, Wang JP. 2019. Mutations in influenza a virus neuraminidase and hemagglutinin confer resistance against a broadly neutralizing hemagglutinin stem antibody. J Virol 93:e01639-18. doi:10.1128/JVI.01639-18 30381484 PMC6321927

[45] Bazzone LE, King M, MacKay CR, Kyawe PP, Meraner P, Lindstrom D, Rojas-Quintero J, Owen CA, Wang JP, Brass AL, Kurt-Jones EA, Finberg RW. 2019. A disintegrin and metalloproteinase 9 domain (ADAM9) is a major susceptibility factor in the early stages of encephalomyocarditis virus infection. MBio 10:e02734-18. doi:10.1128/mBio.02734-18 30723129 PMC6428755

[46] Mor A, White A, Zhang K, Thompson M, Esparza M, Muñoz-Moreno R, Koide K, Lynch KW, García-Sastre A, Fontoura BMA. 2017. Corrigendum: influenza virus mRNA trafficking through host nuclear speckles. Nat Microbiol 2:17002. doi:10.1038/nmicrobiol.2017.2 28067239

[47] Carette JE, Guimaraes CP, Varadarajan M, Park AS, Wuethrich I, Godarova A, Kotecki M, Cochran BH, Spooner E, Ploegh HL, Brummelkamp TR. 2009. Haploid genetic screens in human cells identify host factors used by pathogens. Science 326:1231–1235. doi:10.1126/science.1178955 19965467

[48] Zhao Y, Zou J, Gao Q, Xie S, Cao J, Zhou H. 2021. CMAS and ST3GAL4 play an important role in the adsorption of influenza virus by affecting the synthesis of sialic acid receptors. Int J Mol Sci 22:6081. doi:10.3390/ijms22116081 34200006 PMC8200212

[49] Wong HH, Fung K, Nicholls JM. 2019. MDCK-B4GalNT2 cells disclose A α2,3-sialic acid requirement for the 2009 pandemic H1N1 A/California/04/2009 and NA aid entry of A/WSN/33. Emerg Microbes Infect 8:1428–1437. doi:10.1080/22221751.2019.1665971 31560252 PMC6781475

[50] Sun T, Yu Y, Wu X, Acevedo A, Luo JD, Wang J, Schneider WM, Hurwitz B, Rosenberg BR, Chung H, Rice CM. 2021. Decoupling expression and editing preferences of ADAR1 p150 and p110 isoforms. Proc Natl Acad Sci U S A 118:e2021757118. doi:10.1073/pnas.2021757118 33723056 PMC8000508

[51] Wang JP, Cerny A, Asher DR, Kurt-Jones EA, Bronson RT, Finberg RW. 2010. MDA5 and MAVS mediate type I interferon responses to coxsackie B virus. J Virol 84:254–260. doi:10.1128/JVI.00631-09 19846534 PMC2798442

[52] Nyalwidhe JO, Jurczyk A, Satish B, Redick S, Qaisar N, Trombly MI, Vangala P, Racicot R, Bortell R, Harlan DM, Greiner DL, Brehm MA, Nadler JL, Wang JP. 2020. Proteomic and transcriptional profiles of human stem cell-derived β cells following enteroviral challenge. Microorganisms 8:295. doi:10.3390/microorganisms8020295 32093375 PMC7074978

[53] Gallagher GR, Brehm MA, Finberg RW, Barton BA, Shultz LD, Greiner DL, Bortell R, Wang JP. 2015. Viral infection of engrafted human islets leads to diabetes. Diabetes 64:1358–1369. doi:10.2337/db14-1020 25392246 PMC4375078

[54] Sellmeier M, Weinhold B, Münster-Kühnel A. 2015. CMP-sialic acid synthetase: the point of constriction in the sialylation pathway. Top Curr Chem 366:139–167. doi:10.1007/128_2013_477 24141690

[55] Tenoever BR, Ng S-L, Chua MA, McWhirter SM, García-Sastre A, Maniatis T. 2007. Multiple functions of the IKK-related kinase IKKepsilon in interferon-mediated antiviral immunity. Science 315:1274–1278. doi:10.1126/science.1136567 17332413

[56] García MA, Gil J, Ventoso I, Guerra S, Domingo E, Rivas C, Esteban M. 2006. Impact of protein kinase PKR in cell biology: from antiviral to antiproliferative action. Microbiol Mol Biol Rev 70:1032–1060. doi:10.1128/MMBR.00027-06 17158706 PMC1698511

